# (2*E*)-3-(3-Bromo-4-meth­oxy­phen­yl)-1-(pyridin-2-yl)prop-2-en-1-one

**DOI:** 10.1107/S1600536811000353

**Published:** 2011-01-12

**Authors:** Jerry P. Jasinski, Ray J. Butcher, S. Samshuddin, B. Narayana, H. S. Yathirajan

**Affiliations:** a Department of Chemistry, Keene State College, 229 Main Street, Keene, NH 03435-2001, USA; b Department of Chemistry, Howard University, 525 College Street NW, Washington, DC 20059, USA; c Department of Studies in Chemistry, Mangalore University, Mangalagangotri 574 199, India; d Department of Studies in Chemistry, University of Mysore, Manasagangotri, Mysore 570 006, India

## Abstract

The mean planes of the benzene and pyridine rings in the title compound, C_15_H_12_BrNO_2_, are nearly coplanar, subtending an angle of 2.8 (8)°. The prop-2-en-1-one group is also in the plane of these rings with an N—C—C—O torsion angle of 179.6 (3)°. A weak C—H⋯Br inter­molecular inter­action contributes to the crystal packing, creating a chain-like structure along the *a* axis.

## Related literature

For the pharmacological activity of chalcones, see: Dhar (1981 ▶); Dimmock *et al.* (1999 ▶); Satyanarayana *et al.* (2004 ▶). For their ability to block voltage-dependent potassium channels, see: Yarishkin *et al.* (2008 ▶). For their applications as organic non-linear optical materials due to their SHG conversion efficiency, see: Sarojini *et al.* (2006 ▶) and excellent blue light transmittance and good crystallization ability, see: Goto *et al.* (1991 ▶); Indira *et al.* (2002 ▶); Uchida *et al.* (1998 ▶). For the use of chalcones in the synthesis of various biodynamic heterocyclic compounds such as cyclo­hexenone and pyrazoline derivatives, see: Ashalatha *et al.* (2009 ▶); Sreevidya *et al.* (2010 ▶); Samshuddin *et al.* (2010 ▶); Fun *et al.* (2010*a* ▶,*b* ▶); Jasinski *et al.* (2010*a* ▶,*b* ▶). For the potential use of these compounds or chalcone-rich plant extracts as drugs or food preservatives, see: Di Carlo *et al.* (1999 ▶). For related structures, see: Bibila Mayaya Bisseyou *et al.* (2007 ▶); Liu *et al.* (2005 ▶). 
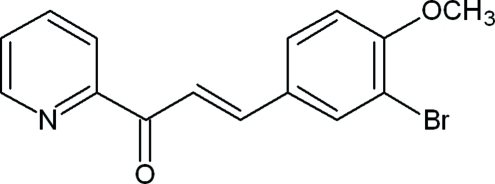


## Experimental

### 

#### Crystal data

C_15_H_12_BrNO_2_
*M* *_r_* = 318.17
Monoclinic, 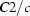

*a* = 26.3402 (13) Å
*b* = 3.8906 (2) Å
*c* = 27.5826 (17) Å
β = 113.892 (5)°
*V* = 2584.4 (2) Å^3^
*Z* = 8
Cu *K*α radiation
μ = 4.31 mm^−1^
*T* = 123 K
0.49 × 0.21 × 0.16 mm

#### Data collection

Oxford Diffraction Xcalibur Ruby Gemini diffractometer
Absorption correction: multi-scan (*CrysAlis RED*; Oxford Diffraction, 2007 ▶) *T*_min_ = 0.535, *T*_max_ = 1.000
3978 measured reflections
2556 independent reflections
2431 reflections with *I* > 2σ(*I*)
*R*_int_ = 0.014

#### Refinement

*R*[*F* ^2^ > 2σ(*F* ^2^)] = 0.040
*wR*(*F* ^2^) = 0.115
*S* = 1.08
2556 reflections
173 parameters
H-atom parameters constrained
Δρ_max_ = 0.85 e Å^−3^
Δρ_min_ = −0.64 e Å^−3^

### 

Data collection: *CrysAlis PRO* (Oxford Diffraction, 2007 ▶); cell refinement: *CrysAlis PRO*; data reduction: *CrysAlis RED* (Oxford Diffraction, 2007 ▶); program(s) used to solve structure: *SHELXS97* (Sheldrick, 2008 ▶); program(s) used to refine structure: *SHELXL97* (Sheldrick, 2008 ▶); molecular graphics: *SHELXTL* (Sheldrick, 2008 ▶); software used to prepare material for publication: *SHELXTL*.

## Supplementary Material

Crystal structure: contains datablocks global, I. DOI: 10.1107/S1600536811000353/ng5098sup1.cif

Structure factors: contains datablocks I. DOI: 10.1107/S1600536811000353/ng5098Isup2.hkl

Additional supplementary materials:  crystallographic information; 3D view; checkCIF report

## Figures and Tables

**Table 1:** Hydrogen-bond geometry (Å, °)

*D*—H⋯*A*	*D*—H	H⋯*A*	*D*⋯*A*	*D*—H⋯*A*
C3—H3*A*⋯Br1^i^	0.95	3.04	3.870 (3)	146

Symmetry code: (i) 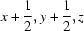.

**Fig. 1.**
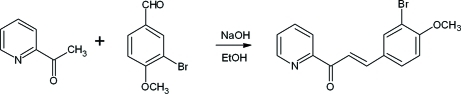
REaction scheme for C15H12BrNO2.

**Fig. 2.**
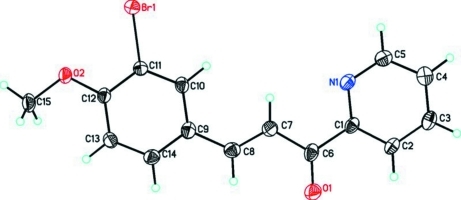
Molecular structure of the title compound showing the atom labeling scheme and 50% probability displacement ellipsoids.

**Fig. 3.**
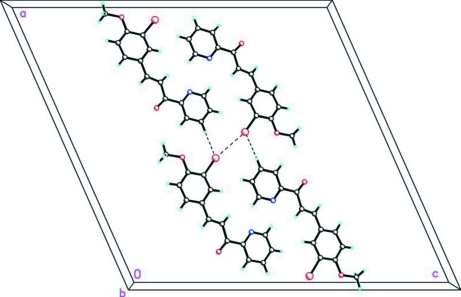
Packing diagram of the title compound viewed down the b axis. Dashed lines indicate weak C—H···Br intermolecular hydrogen bond interactions creating a 2-D network structure along [101].

## supplementary crystallographic information

### Comment

Chalcones constitute an important family of substances belonging to flavonoids, a large group of natural and synthetic products with interesting physicochemical properties, biological activity and structural characteristics. Chalcones are highly reactive substances of varied nature.  They have been reported to possess many interesting pharmacological activities (Dhar, 1981) including anti-inflammatory, antimicrobial, antifungal, antioxidant, cytotoxic, antitumor and anticancer activities (Dimmock *et al.*, 1999; Satyanarayana *et al.*, 2004). Some chalcones demonstrated the ability to block voltage-dependent potassium channels (Yarishkin *et al.*, 2008). Chalcones are also finding application as organic nonlinear optical materials (NLO) for their SHG conversion efficiency (Sarojini *et al.*, 2006). Among several organic compounds reported which have NLO properties, chalcone derivatives are a recognized material because of their excellent blue light transmittance and good crystallization ability (Goto *et al.*,1991; Uchida *et al.*,1998; Indira *et al.*, 2002). The basic skeleton of chalcones which possess α,β-unsaturated carbonyl group is useful as the starting material for the synthesis of various biodynamic heterocyclic compounds such as cyclohexenone derivatives and pyrazoline derivatives (Ashalatha *et al.*, 2009; Sreevidya *et al.*, 2010; Samshuddin *et al.*, 2010; Fun *et al.*, 2010*a*,*b*; Jasinski *et al.*, 2010*a*,*b*). The radical quenching properties of the phenolic groups present in many chalcones have raised interest in using these compounds or chalcone rich plant extracts as drugs or food preservatives (Di Carlo *et al.*, 1999). The crystal  structures of some chalcones derived from acetyl pyridine  viz., (Z)-3-(2,6-dichlorophenyl)-1-(pyridin-3-yl)-2- (1H-1,2,4-triazol-1-yl)prop-2-en-1-one (Liu *et al.*, 2005), 3-(3-chlorophenyl)-1-(2-methylimidazo[1,2-a]pyridin-3-yl)prop-2-en-1-one (Bibila Mayaya Bisseyou *et al.*, 2007) have been reported. In continuation of our studies on chalcones and their derivatives, the title compound (I) was prepared and its crystal structure is reported.

The mean planes of the benzene and pyridine rings in the title compound, C_15_H_12_BrNO_2_, are nearly planar being separated by only 2.8 (8)° (Fig. 2).  The prop-2-en-1-one group is also in the plane of these rings with a N1-C1-C6-O1 torsion angle of 179.6 (3)°.  A weak C—H···Br intermolecular interaction (Table 1) contributes to crystal packing creating a 2-D network structure along [101].(Fig. 3).

### Experimental

To a mixture of 2-acetyl pyridine (1.21 g, 0.01 mol) and 3-bromo-4-methoxybenzaldehyde (2.15 g, 0.01 mol) in 30 ml e thanol, 10 ml of 10% sodium hydroxide solution was added and stirred at 5–10°C for 3 h (Fig. 1). The precipitate formed was collected by filtration and purified by recrystallization from ethanol. The single-crystal was grown from acetonitrile by slow evaporation method and yield of the compound was 82%. (m.p. 428 K). Analytical data: Found (Cald): C %: 56.58(56.62); H%: 3.78 (3.80); N%: 4.37 (4.40).

### Refinement

All of the H atoms were placed in their calculated positions and then refined using the riding model with Atom—H lengths of 0.95Å (CH), or 0.98Å (CH_3_). Isotropic displacement parameters for these atoms were set to 1.18–1.20 (CH) or 1.49 (CH_3_) times *U*_eq_ of the parent atom.

### Crystal data

**Table:**

C_15_H_12_BrNO_2_	*F*(000) = 1280
*M**_r_* = 318.17	*D*_x_ = 1.635 Mg m^−^^3^
Monoclinic, *C*2/*c*	Cu *K*α radiation, λ = 1.54178 Å
Hall symbol: -C 2yc	Cell parameters from 3510 reflections
*a* = 26.3402 (13) Å	θ = 4.7–74.0°
*b* = 3.8906 (2) Å	µ = 4.31 mm^−^^1^
*c* = 27.5826 (17) Å	*T* = 123 K
β = 113.892 (5)°	Needle, colorless
*V* = 2584.4 (2) Å^3^	0.49 × 0.21 × 0.16 mm
*Z* = 8	

### Data collection

**Table:**

Oxford Diffraction Xcalibur Ruby Gemini diffractometer	2556 independent reflections
Radiation source: Enhance (Cu) X-ray Source	2431 reflections with *I* > 2σ(*I*)
graphite	*R*_int_ = 0.014
Detector resolution: 10.5081 pixels mm^-1^	θ_max_ = 74.1°, θ_min_ = 6.0°
ω scans	*h* = −29→32
Absorption correction: multi-scan (*CrysAlis RED*; Oxford Diffraction, 2007)	*k* = −4→2
*T*_min_ = 0.535, *T*_max_ = 1.000	*l* = −34→33
3978 measured reflections	

### Refinement

**Table:**

Refinement on *F*^2^	Primary atom site location: structure-invariant direct methods
Least-squares matrix: full	Secondary atom site location: difference Fourier map
*R*[*F*^2^ > 2σ(*F*^2^)] = 0.040	Hydrogen site location: inferred from neighbouring sites
*wR*(*F*^2^) = 0.115	H-atom parameters constrained
*S* = 1.08	*w* = 1/[σ^2^(*F*_o_^2^) + (0.0704*P*)^2^ + 6.6225*P*] where *P* = (*F*_o_^2^ + 2*F*_c_^2^)/3
2556 reflections	(Δ/σ)_max_ < 0.001
173 parameters	Δρ_max_ = 0.85 e Å^−^^3^
0 restraints	Δρ_min_ = −0.64 e Å^−^^3^

### Special details

**Table:**

Geometry. All e.s.d.'s (except the e.s.d. in the dihedral angle between two l.s. planes) are estimated using the full covariance matrix. The cell e.s.d.'s are taken into account individually in the estimation of e.s.d.'s in distances, angles and torsion angles; correlations between e.s.d.'s in cell parameters are only used when they are defined by crystal symmetry. An approximate (isotropic) treatment of cell e.s.d.'s is used for estimating e.s.d.'s involving l.s. planes.
Refinement. Refinement of *F*^2^ against ALL reflections. The weighted *R*-factor *wR* and goodness of fit *S* are based on *F*^2^, conventional *R*-factors *R* are based on *F*, with *F* set to zero for negative *F*^2^. The threshold expression of *F*^2^ > σ(*F*^2^) is used only for calculating *R*-factors(gt) *etc*. and is not relevant to the choice of reflections for refinement. *R*-factors based on *F*^2^ are statistically about twice as large as those based on *F*, and *R*- factors based on ALL data will be even larger.

### Fractional atomic coordinates and isotropic or equivalent isotropic displacement parameters (Å^2^)

**Table:**

	*x*	*y*	*z*	*U*_iso_*/*U*_eq_	
Br1	0.046034 (12)	0.06121 (9)	0.063358 (11)	0.03852 (15)	
O1	0.36895 (11)	0.4707 (8)	0.17800 (10)	0.0507 (6)	
O2	0.04309 (8)	0.3262 (7)	0.16281 (8)	0.0431 (5)	
N1	0.30803 (11)	0.0395 (8)	0.05431 (11)	0.0410 (6)	
C1	0.34866 (11)	0.1894 (9)	0.09571 (12)	0.0359 (6)	
C2	0.40303 (12)	0.2175 (8)	0.10013 (13)	0.0382 (6)	
H2A	0.4308	0.3251	0.1300	0.046*	
C3	0.41559 (13)	0.0837 (9)	0.05953 (15)	0.0441 (8)	
H3A	0.4523	0.0956	0.0614	0.053*	
C4	0.37382 (15)	−0.0664 (9)	0.01663 (15)	0.0451 (8)	
H4A	0.3812	−0.1569	−0.0119	0.054*	
C5	0.32078 (15)	−0.0833 (9)	0.01567 (14)	0.0442 (8)	
H5A	0.2922	−0.1875	−0.0140	0.053*	
C6	0.33420 (13)	0.3330 (10)	0.13914 (12)	0.0420 (7)	
C7	0.27542 (13)	0.3026 (9)	0.13151 (12)	0.0410 (7)	
H7A	0.2503	0.1747	0.1023	0.049*	
C8	0.25712 (14)	0.4531 (9)	0.16526 (13)	0.0404 (7)	
H8A	0.2833	0.5857	0.1931	0.048*	
C9	0.20072 (13)	0.4324 (9)	0.16324 (12)	0.0389 (7)	
C10	0.15685 (12)	0.2790 (9)	0.12093 (11)	0.0359 (6)	
H10A	0.1628	0.1909	0.0915	0.043*	
C11	0.10531 (11)	0.2558 (7)	0.12193 (11)	0.0311 (6)	
C12	0.09521 (12)	0.3743 (8)	0.16536 (11)	0.0335 (6)	
C13	0.13844 (13)	0.5282 (9)	0.20707 (13)	0.0374 (7)	
H13A	0.1327	0.6135	0.2367	0.045*	
C14	0.19004 (14)	0.5571 (9)	0.20544 (13)	0.0395 (7)	
H14A	0.2192	0.6660	0.2341	0.047*	
C15	0.03317 (15)	0.4373 (11)	0.20775 (14)	0.0489 (9)	
H15A	−0.0049	0.3784	0.2024	0.073*	
H15B	0.0383	0.6868	0.2118	0.073*	
H15C	0.0594	0.3227	0.2398	0.073*	

### Atomic displacement parameters (Å^2^)

**Table:**

	*U*^11^	*U*^22^	*U*^33^	*U*^12^	*U*^13^	*U*^23^
Br1	0.0377 (2)	0.0436 (2)	0.0325 (2)	−0.00132 (12)	0.01247 (15)	−0.00231 (12)
O1	0.0427 (13)	0.0683 (16)	0.0394 (13)	−0.0095 (12)	0.0149 (11)	−0.0008 (11)
O2	0.0317 (10)	0.0681 (15)	0.0339 (10)	0.0021 (11)	0.0177 (8)	−0.0024 (11)
N1	0.0317 (13)	0.0494 (16)	0.0400 (14)	−0.0043 (11)	0.0124 (11)	0.0067 (12)
C1	0.0283 (13)	0.0413 (16)	0.0366 (14)	−0.0025 (12)	0.0116 (11)	0.0114 (13)
C2	0.0291 (13)	0.0392 (16)	0.0450 (16)	−0.0025 (12)	0.0136 (12)	0.0083 (13)
C3	0.0343 (16)	0.0416 (19)	0.060 (2)	0.0050 (13)	0.0231 (15)	0.0099 (15)
C4	0.0501 (19)	0.0388 (18)	0.0502 (19)	0.0078 (14)	0.0241 (16)	0.0077 (14)
C5	0.0418 (17)	0.0439 (19)	0.0430 (17)	−0.0038 (13)	0.0130 (14)	0.0025 (14)
C6	0.0341 (14)	0.0554 (19)	0.0385 (16)	0.0013 (14)	0.0168 (13)	0.0130 (15)
C7	0.0378 (15)	0.0466 (18)	0.0381 (15)	−0.0029 (14)	0.0149 (12)	−0.0003 (14)
C8	0.0409 (16)	0.0433 (18)	0.0346 (15)	−0.0030 (13)	0.0130 (13)	0.0018 (13)
C9	0.0340 (15)	0.0511 (19)	0.0322 (15)	0.0061 (13)	0.0141 (12)	0.0104 (13)
C10	0.0342 (13)	0.0466 (17)	0.0288 (13)	0.0082 (13)	0.0147 (11)	0.0069 (12)
C11	0.0319 (13)	0.0315 (14)	0.0289 (12)	0.0025 (11)	0.0113 (10)	0.0037 (11)
C12	0.0301 (13)	0.0391 (15)	0.0325 (14)	0.0051 (12)	0.0140 (11)	0.0040 (12)
C13	0.0385 (16)	0.0416 (16)	0.0326 (14)	0.0069 (13)	0.0149 (13)	0.0001 (12)
C14	0.0354 (15)	0.0464 (18)	0.0322 (15)	0.0004 (13)	0.0092 (12)	0.0028 (13)
C15	0.0409 (17)	0.074 (3)	0.0398 (17)	0.0117 (16)	0.0243 (15)	0.0033 (16)

### Geometric parameters (Å, °)

**Table:**

Br1—C11	1.892 (3)	C7—C8	1.344 (5)
O1—C6	1.216 (4)	C7—H7A	0.9500
O2—C12	1.359 (3)	C8—C9	1.466 (4)
O2—C15	1.433 (4)	C8—H8A	0.9500
N1—C5	1.329 (5)	C9—C14	1.391 (5)
N1—C1	1.341 (4)	C9—C10	1.400 (5)
C1—C2	1.391 (4)	C10—C11	1.372 (4)
C1—C6	1.504 (5)	C10—H10A	0.9500
C2—C3	1.391 (5)	C11—C12	1.406 (4)
C2—H2A	0.9500	C12—C13	1.385 (4)
C3—C4	1.377 (5)	C13—C14	1.383 (5)
C3—H3A	0.9500	C13—H13A	0.9500
C4—C5	1.388 (5)	C14—H14A	0.9500
C4—H4A	0.9500	C15—H15A	0.9800
C5—H5A	0.9500	C15—H15B	0.9800
C6—C7	1.480 (4)	C15—H15C	0.9800
			
C12—O2—C15	116.6 (3)	C9—C8—H8A	116.8
C5—N1—C1	117.7 (3)	C14—C9—C10	117.9 (3)
N1—C1—C2	123.1 (3)	C14—C9—C8	119.6 (3)
N1—C1—C6	117.9 (3)	C10—C9—C8	122.4 (3)
C2—C1—C6	119.0 (3)	C11—C10—C9	120.0 (3)
C3—C2—C1	118.2 (3)	C11—C10—H10A	120.0
C3—C2—H2A	120.9	C9—C10—H10A	120.0
C1—C2—H2A	120.9	C10—C11—C12	121.7 (3)
C4—C3—C2	118.9 (3)	C10—C11—Br1	119.4 (2)
C4—C3—H3A	120.6	C12—C11—Br1	118.9 (2)
C2—C3—H3A	120.6	O2—C12—C13	125.2 (3)
C3—C4—C5	118.9 (3)	O2—C12—C11	116.5 (3)
C3—C4—H4A	120.5	C13—C12—C11	118.3 (3)
C5—C4—H4A	120.5	C14—C13—C12	119.8 (3)
N1—C5—C4	123.2 (3)	C14—C13—H13A	120.1
N1—C5—H5A	118.4	C12—C13—H13A	120.1
C4—C5—H5A	118.4	C13—C14—C9	122.2 (3)
O1—C6—C7	122.1 (3)	C13—C14—H14A	118.9
O1—C6—C1	121.5 (3)	C9—C14—H14A	118.9
C7—C6—C1	116.4 (3)	O2—C15—H15A	109.5
C8—C7—C6	121.0 (3)	O2—C15—H15B	109.5
C8—C7—H7A	119.5	H15A—C15—H15B	109.5
C6—C7—H7A	119.5	O2—C15—H15C	109.5
C7—C8—C9	126.3 (3)	H15A—C15—H15C	109.5
C7—C8—H8A	116.8	H15B—C15—H15C	109.5
			
C5—N1—C1—C2	−0.8 (5)	C7—C8—C9—C10	−7.5 (5)
C5—N1—C1—C6	179.2 (3)	C14—C9—C10—C11	0.1 (5)
N1—C1—C2—C3	0.1 (5)	C8—C9—C10—C11	177.7 (3)
C6—C1—C2—C3	−179.9 (3)	C9—C10—C11—C12	−1.8 (5)
C1—C2—C3—C4	0.8 (5)	C9—C10—C11—Br1	178.0 (2)
C2—C3—C4—C5	−0.9 (5)	C15—O2—C12—C13	−1.6 (5)
C1—N1—C5—C4	0.7 (5)	C15—O2—C12—C11	178.1 (3)
C3—C4—C5—N1	0.1 (5)	C10—C11—C12—O2	−177.6 (3)
N1—C1—C6—O1	179.6 (3)	Br1—C11—C12—O2	2.7 (4)
C2—C1—C6—O1	−0.4 (5)	C10—C11—C12—C13	2.2 (5)
N1—C1—C6—C7	−1.4 (4)	Br1—C11—C12—C13	−177.6 (2)
C2—C1—C6—C7	178.6 (3)	O2—C12—C13—C14	178.9 (3)
O1—C6—C7—C8	5.8 (6)	C11—C12—C13—C14	−0.9 (5)
C1—C6—C7—C8	−173.2 (3)	C12—C13—C14—C9	−0.8 (5)
C6—C7—C8—C9	−177.7 (3)	C10—C9—C14—C13	1.2 (5)
C7—C8—C9—C14	170.1 (3)	C8—C9—C14—C13	−176.5 (3)

### Hydrogen-bond geometry (Å, °)

**Table:**

*D*—H···*A*	*D*—H	H···*A*	*D*···*A*	*D*—H···*A*
C3—H3A···Br1^i^	0.95	3.04	3.870 (3)	146

Symmetry codes: (i) *x*+1/2, *y*+1/2, *z*.
